# Supplementary material for: HMGA2 as a prognostic and immune biomarker in hepatocellular carcinoma: Comprehensive analysis of the HMG family and experiments validation
Source: PLoS One. 2024 Nov 26;19(11):e0311204. doi: 10.1371/journal.pone.0311204 (PMC11594397; doi:10.1371/journal.pone.0311204)
Supplement: S1 Table — (DOCX) [file pone.0311204.s001.docx]

**S1 Table. Univariate and multivariate analyses of clinicopathological variables and HMGs expressions for prediction of OS of TCGA patients.**

| Characteristics | Total(N) | Univariate analysis | |  | Multivariate analysis | |
| --- | --- | --- | --- | --- | --- | --- |
|  |  | Hazard ratio (95% CI) | P value |  | Hazard ratio (95% CI) | P value |
| HMGA1 | 373 |  |  |  |  |  |
| Low | 187 | Reference |  |  | Reference |  |
| High | 186 | 1.827 (1.287 - 2.593) | **< 0.001** |  | 1.650 (0.998 - 2.729) | 0.051 |
| HMGA2 | 373 |  |  |  |  |  |
| Low | 186 | Reference |  |  | Reference |  |
| High | 187 | 1.366 (0.967 - 1.928) | 0.077 |  | 1.114 (0.691 - 1.796) | 0.658 |
| HMGB1 | 373 |  |  |  |  |  |
| Low | 187 | Reference |  |  |  |  |
| High | 186 | 1.301 (0.920 - 1.840) | 0.137 |  |  |  |
| HMGB2 | 373 |  |  |  |  |  |
| Low | 187 | Reference |  |  | Reference |  |
| High | 186 | 1.539 (1.087 - 2.180) | **0.015** |  | 1.352 (0.753 - 2.427) | 0.313 |
| HMGB3 | 373 |  |  |  |  |  |
| Low | 187 | Reference |  |  | Reference |  |
| High | 186 | 1.382 (0.976 - 1.956) | 0.068 |  | 1.093 (0.696 - 1.716) | 0.698 |
| HMGN1 | 373 |  |  |  |  |  |
| Low | 187 | Reference |  |  | Reference |  |
| High | 186 | 1.426 (1.007 - 2.018) | **0.046** |  | 0.878 (0.523 - 1.477) | 0.625 |
| HMGN2 | 373 |  |  |  |  |  |
| Low | 187 | Reference |  |  | Reference |  |
| High | 186 | 1.599 (1.128 - 2.267) | **0.008** |  | 1.155 (0.679 - 1.965) | 0.596 |
| HMGN3 | 373 |  |  |  |  |  |
| Low | 187 | Reference |  |  |  |  |
| High | 186 | 1.272 (0.900 - 1.797) | 0.173 |  |  |  |
| HMGN4 | 373 |  |  |  |  |  |
| Low | 187 | Reference |  |  | Reference |  |
| High | 186 | 1.615 (1.140 - 2.286) | **0.007** |  | 1.037 (0.601 - 1.789) | 0.897 |
| HMGN5 | 373 |  |  |  |  |  |
| Low | 186 | Reference |  |  |  |  |
| High | 187 | 1.158 (0.820 - 1.635) | 0.405 |  |  |  |
| Pathologic T stage | 370 |  |  |  |  |  |
| T1 | 183 | Reference |  |  | Reference |  |
| T2&T3&T4 | 187 | 2.126 (1.481 - 3.052) | **< 0.001** |  | 0.737 (0.094 - 5.783) | 0.772 |
| Pathologic M stage | 272 |  |  |  |  |  |
| M0 | 268 | Reference |  |  | Reference |  |
| M1 | 4 | 4.077 (1.281 - 12.973) | **0.017** |  | 3.597 (1.019 - 12.701) | **0.047** |
| Pathologic stage | 349 |  |  |  |  |  |
| Stage I | 173 | Reference |  |  | Reference |  |
| Stage II&Stage III&Stage IV | 176 | 2.090 (1.429 - 3.055) | **< 0.001** |  | 2.887 (0.357 - 23.350) | 0.320 |
| AFP(ng/ml) | 279 |  |  |  |  |  |
| <= 400 | 215 | Reference |  |  |  |  |
| > 400 | 64 | 1.075 (0.658 - 1.759) | 0.772 |  |  |  |
| Albumin(g/dl) | 299 |  |  |  |  |  |
| < 3.5 | 69 | Reference |  |  |  |  |
| >= 3.5 | 230 | 0.897 (0.549 - 1.464) | 0.662 |  |  |  |
| Child-Pugh grade | 240 |  |  |  |  |  |
| A | 218 | Reference |  |  |  |  |
| B&C | 22 | 1.643 (0.811 - 3.330) | 0.168 |  |  |  |
