# Supplementary material for: HMGA2 as a prognostic and immune biomarker in hepatocellular carcinoma: Comprehensive analysis of the HMG family and experiments validation
Source: PLoS One. 2024 Nov 26;19(11):e0311204. doi: 10.1371/journal.pone.0311204 (PMC11594397; doi:10.1371/journal.pone.0311204)
Supplement: S2 Table — (DOCX) [file pone.0311204.s002.docx]

**S2 Table. Univariate and multivariate analyses of clinicopathological variables and HMGs expressions for prediction of DSS of TCGA patients.**

| Characteristics | Total(N) | Univariate analysis | |  | Multivariate analysis | |
| --- | --- | --- | --- | --- | --- | --- |
|  |  | Hazard ratio (95% CI) | P value |  | Hazard ratio (95% CI) | P value |
| HMGA1 | 365 |  |  |  |  |  |
| Low | 187 | Reference |  |  |  |  |
| High | 178 | 1.438 (0.924 - 2.237) | 0.107 |  |  |  |
| HMGA2 | 365 |  |  |  |  |  |
| Low | 181 | Reference |  |  | Reference |  |
| High | 184 | 1.652 (1.058 - 2.581) | **0.027** |  | 0.759 (0.325 - 1.771) | 0.523 |
| HMGB1 | 365 |  |  |  |  |  |
| Low | 181 | Reference |  |  |  |  |
| High | 184 | 1.403 (0.896 - 2.195) | 0.139 |  |  |  |
| HMGB2 | 365 |  |  |  |  |  |
| Low | 183 | Reference |  |  | Reference |  |
| High | 182 | 1.627 (1.040 - 2.545) | **0.033** |  | 2.759 (1.013 - 7.519) | **0.047** |
| HMGB3 | 365 |  |  |  |  |  |
| Low | 185 | Reference |  |  |  |  |
| High | 180 | 1.333 (0.855 - 2.079) | 0.205 |  |  |  |
| HMGN1 | 365 |  |  |  |  |  |
| Low | 183 | Reference |  |  | Reference |  |
| High | 182 | 1.715 (1.090 - 2.699) | **0.020** |  | 0.924 (0.397 - 2.152) | 0.855 |
| HMGN2 | 365 |  |  |  |  |  |
| Low | 183 | Reference |  |  | Reference |  |
| High | 182 | 1.720 (1.096 - 2.698) | **0.018** |  | 0.781 (0.310 - 1.962) | 0.598 |
| HMGN3 | 365 |  |  |  |  |  |
| Low | 184 | Reference |  |  |  |  |
| High | 181 | 1.345 (0.863 - 2.096) | 0.191 |  |  |  |
| HMGN4 | 365 |  |  |  |  |  |
| Low | 182 | Reference |  |  | Reference |  |
| High | 183 | 1.835 (1.170 - 2.877) | **0.008** |  | 1.123 (0.462 - 2.734) | 0.798 |
| HMGN5 | 365 |  |  |  |  |  |
| Low | 181 | Reference |  |  |  |  |
| High | 184 | 1.368 (0.874 - 2.139) | 0.170 |  |  |  |
| Pathologic T stage | 362 |  |  |  |  |  |
| T1 | 180 | Reference |  |  | Reference |  |
| T2&T3&T4 | 182 | 2.829 (1.747 - 4.582) | **< 0.001** |  | 1.287 (0.108 - 15.355) | 0.842 |
| Pathologic M stage | 268 |  |  |  |  |  |
| M0 | 265 | Reference |  |  | Reference |  |
| M1 | 3 | 5.166 (1.246 - 21.430) | **0.024** |  | 11.855 (2.178 - 64.545) | **0.004** |
| Pathologic stage | 341 |  |  |  |  |  |
| Stage I | 170 | Reference |  |  | Reference |  |
| Stage II&Stage III&Stage IV | 171 | 2.909 (1.718 - 4.925) | **< 0.001** |  | 1.603 (0.137 - 18.740) | 0.707 |
| AFP(ng/ml) | 275 |  |  |  |  |  |
| <= 400 | 214 | Reference |  |  |  |  |
| > 400 | 61 | 0.867 (0.450 - 1.668) | 0.668 |  |  |  |
| Albumin(g/dl) | 294 |  |  |  |  |  |
| < 3.5 | 67 | Reference |  |  |  |  |
| >= 3.5 | 227 | 1.148 (0.586 - 2.250) | 0.687 |  |  |  |
| Child-Pugh grade | 235 |  |  |  |  |  |
| A | 214 | Reference |  |  | Reference |  |
| B&C | 21 | 2.560 (1.123 - 5.834) | **0.025** |  | 3.667 (1.319 - 10.194) | **0.013** |
