# Supplementary material for: HMGA2 as a prognostic and immune biomarker in hepatocellular carcinoma: Comprehensive analysis of the HMG family and experiments validation
Source: PLoS One. 2024 Nov 26;19(11):e0311204. doi: 10.1371/journal.pone.0311204 (PMC11594397; doi:10.1371/journal.pone.0311204)
Supplement: S3 Table — (DOCX) [file pone.0311204.s003.docx]

**S3 Table. Univariate and multivariate analyses of clinicopathological variables and HMGs expressions for prediction of PFI of TCGA patients.**

| Characteristics | Total(N) | Univariate analysis | |  | Multivariate analysis | |
| --- | --- | --- | --- | --- | --- | --- |
|  |  | Hazard ratio (95% CI) | P value |  | Hazard ratio (95% CI) | P value |
| HMGA1 | 373 |  |  |  |  |  |
| Low | 187 | Reference |  |  | Reference |  |
| High | 186 | 1.457 (1.089 - 1.949) | **0.011** |  | 1.172 (0.783 - 1.753) | 0.440 |
| HMGA2 | 373 |  |  |  |  |  |
| Low | 186 | Reference |  |  |  |  |
| High | 187 | 1.211 (0.905 - 1.621) | 0.197 |  |  |  |
| HMGB1 | 373 |  |  |  |  |  |
| Low | 187 | Reference |  |  |  |  |
| High | 186 | 1.205 (0.901 - 1.612) | 0.208 |  |  |  |
| HMGB2 | 373 |  |  |  |  |  |
| Low | 187 | Reference |  |  | Reference |  |
| High | 186 | 1.600 (1.195 - 2.142) | **0.002** |  | 1.530 (0.971 - 2.411) | 0.067 |
| HMGB3 | 373 |  |  |  |  |  |
| Low | 187 | Reference |  |  |  |  |
| High | 186 | 1.023 (0.765 - 1.367) | 0.880 |  |  |  |
| HMGN1 | 373 |  |  |  |  |  |
| Low | 187 | Reference |  |  | Reference |  |
| High | 186 | 1.663 (1.241 - 2.228) | **< 0.001** |  | 1.145 (0.748 - 1.752) | 0.533 |
| HMGN2 | 373 |  |  |  |  |  |
| Low | 187 | Reference |  |  | Reference |  |
| High | 186 | 1.296 (0.969 - 1.732) | 0.080 |  | 0.816 (0.535 - 1.246) | 0.347 |
| HMGN3 | 373 |  |  |  |  |  |
| Low | 187 | Reference |  |  | Reference |  |
| High | 186 | 1.379 (1.031 - 1.844) | **0.030** |  | 1.224 (0.849 - 1.763) | 0.279 |
| HMGN4 | 373 |  |  |  |  |  |
| Low | 187 | Reference |  |  | Reference |  |
| High | 186 | 1.437 (1.075 - 1.921) | **0.014** |  | 0.932 (0.600 - 1.448) | 0.754 |
| HMGN5 | 373 |  |  |  |  |  |
| Low | 186 | Reference |  |  |  |  |
| High | 187 | 1.043 (0.780 - 1.394) | 0.778 |  |  |  |
| Pathologic T stage | 370 |  |  |  |  |  |
| T1 | 183 | Reference |  |  | Reference |  |
| T2&T3&T4 | 187 | 2.360 (1.745 - 3.191) | **< 0.001** |  | 0.667 (0.088 - 5.033) | 0.694 |
| Pathologic M stage | 272 |  |  |  |  |  |
| M0 | 268 | Reference |  |  | Reference |  |
| M1 | 4 | 3.476 (1.091 - 11.076) | **0.035** |  | 3.009 (0.909 - 9.963) | 0.071 |
| Pathologic stage | 349 |  |  |  |  |  |
| Stage I | 173 | Reference |  |  | Reference |  |
| Stage II&Stage III&Stage IV | 176 | 2.284 (1.670 - 3.122) | **< 0.001** |  | 2.706 (0.354 - 20.700) | 0.338 |
| AFP(ng/ml) | 279 |  |  |  |  |  |
| <= 400 | 215 | Reference |  |  |  |  |
| > 400 | 64 | 1.045 (0.698 - 1.563) | 0.832 |  |  |  |
| Albumin(g/dl) | 299 |  |  |  |  |  |
| < 3.5 | 69 | Reference |  |  |  |  |
| >= 3.5 | 230 | 0.911 (0.618 - 1.341) | 0.636 |  |  |  |
| Child-Pugh grade | 240 |  |  |  |  |  |
| A | 218 | Reference |  |  |  |  |
| B&C | 22 | 1.395 (0.765 - 2.545) | 0.277 |  |  |  |
