# Supplementary figures and images for: HMGA2 as a prognostic and immune biomarker in hepatocellular carcinoma: Comprehensive analysis of the HMG family and experiments validation
Source: PLoS One. 2024 Nov 26;19(11):e0311204. doi: 10.1371/journal.pone.0311204 (PMC11594397; doi:10.1371/journal.pone.0311204)

**S1 Fig. ROC curves were plotted to validate the diagnostic efficacy of HMGs using GSE84402 and GSE76427.**

**
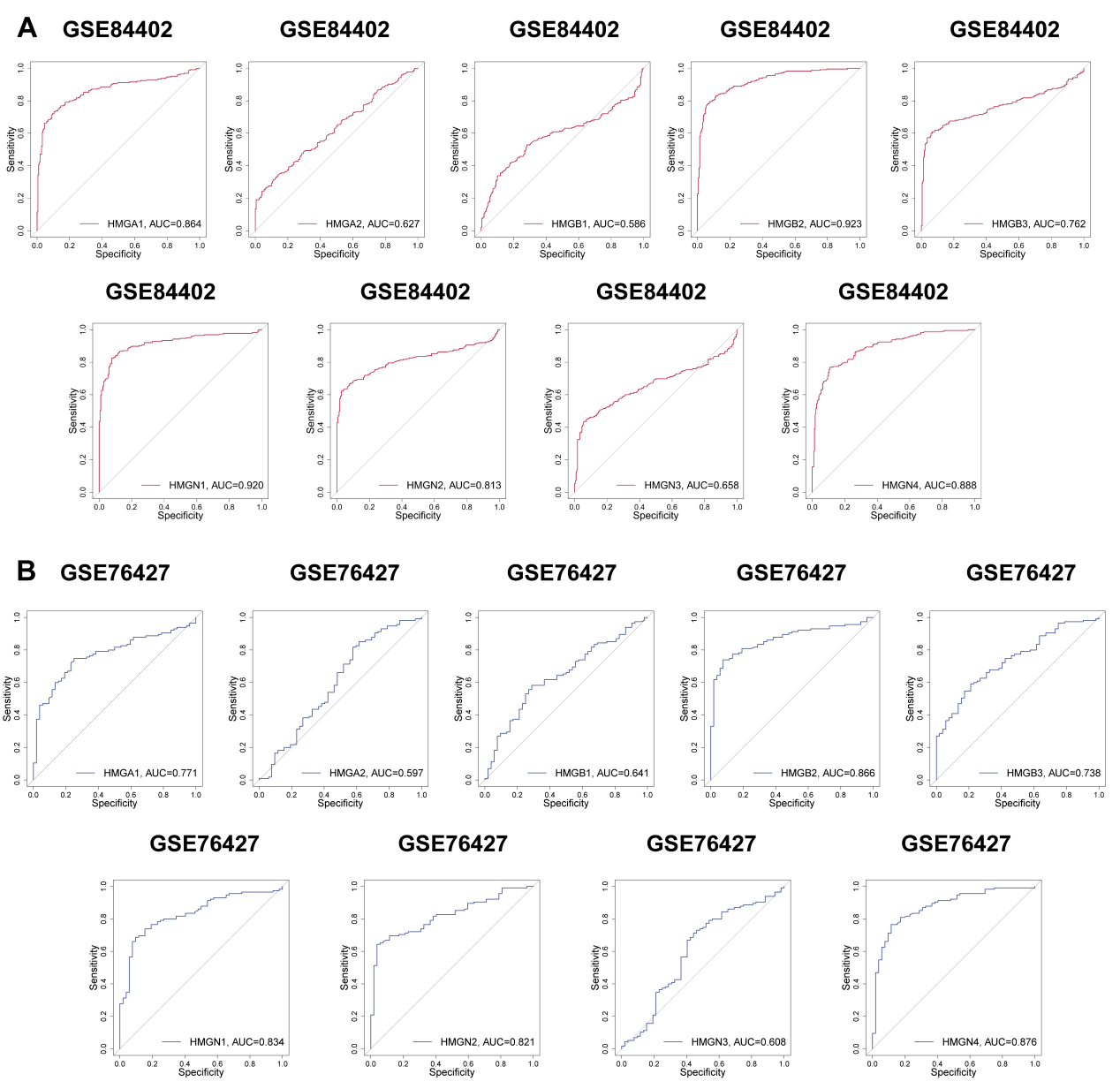
**

Supplement: S1 Fig — (DOCX) [file pone.0311204.s004.docx]

**The original uncropped images underlying all blot results.**

**Fig 12A**


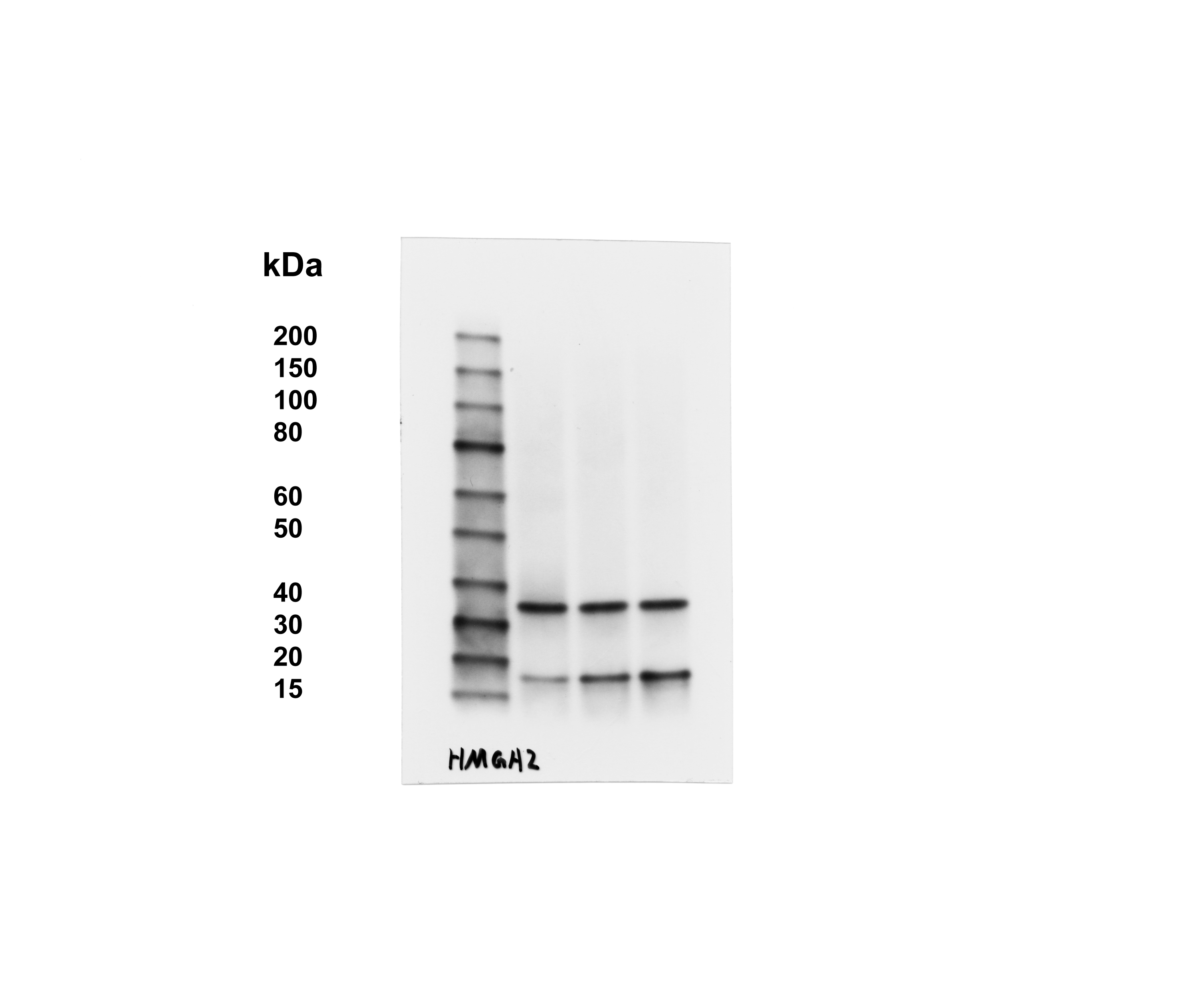


**Fig 12B**


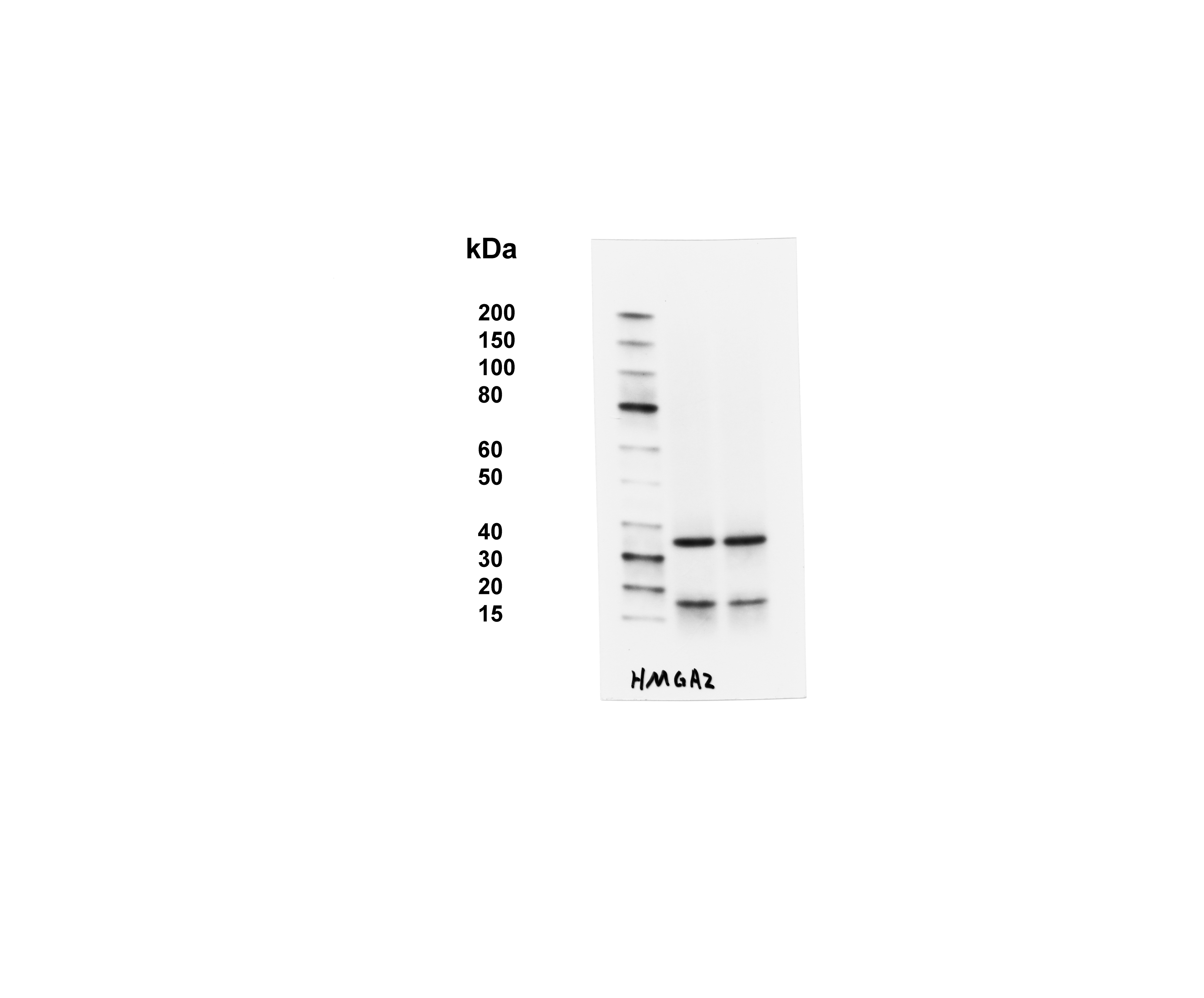


**Fig 12C**


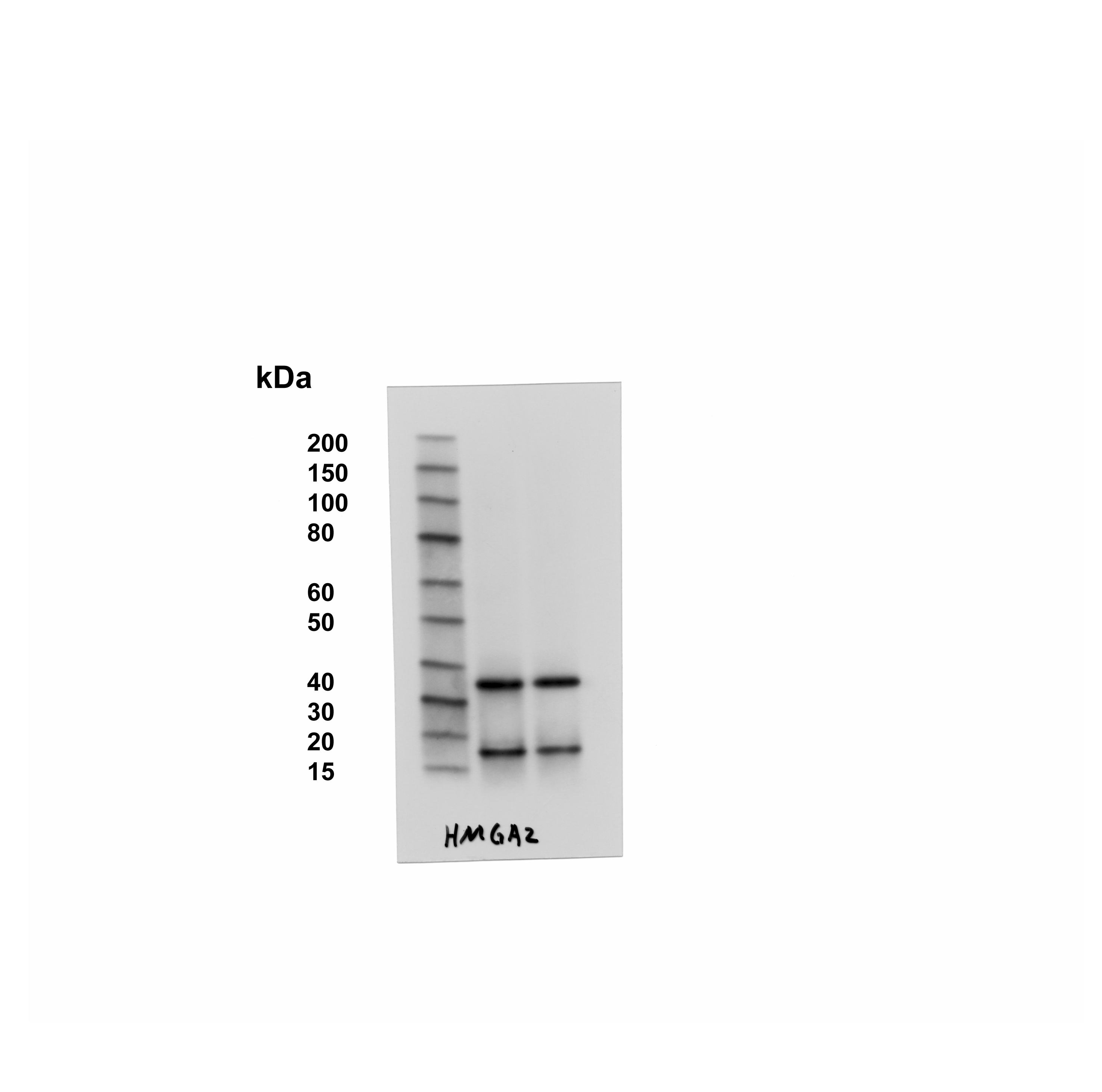

Supplement: S1 File — (DOCX) [file pone.0311204.s005.docx]
